# *Ziziphus* *jujuba* “Huizao” Polysaccharides Exert Immunomodulatory Activity In Vitro and In Vivo by Modulating the TLR4/MAPK/NF-κB Signalling Pathway

**DOI:** 10.3390/foods15020292

**Published:** 2026-01-13

**Authors:** Bin Li, Ting Yang, Jingteng Wang, Xin Shang, Ruxianguli Maimaitiyiming, Jun Xing, Bin Wu, Yinghua Fu

**Affiliations:** 1 College of Smart Agriculture (Research Institute), Xinjiang University, Urumqi 830017, China; 18242180424@163.com (B.L.); xjuyt6122@163.com (T.Y.); 18769520685@163.com (J.W.); sshangxin0919@163.com (X.S.); rshngl@163.com (R.M.); xinjung@163.com (J.X.); 2 Institute of Agro-Products Storage and Processing, Xinjiang Academy of Agricultural Sciences, Urumqi 830091, China; xjuwubin0320@sina.com

**Keywords:** “*Huizao*” polysaccharides, regulating immune response, macrophages, immunosuppressed mice, cytokines, oxidative stress

## Abstract

*Ziziphus jujuba* is an important source of polysaccharides in food supply, and studies have demonstrated that polysaccharides serve as the principal active constituents responsible for immunomodulatory effects. The results indicated that “*Huizao*” polysaccharides (HP2-1) increased the viability and phagocytic activity of RAW264.7 cells and triggered immune responses by promoting cytokines TNF-α, IL-6, and IL-1β secretion, as well as NO and ROS production. In addition, HP2-1 also stimulated cytokine production, elevated immunoglobulin levels, and alleviated oxidative stress via increasing antioxidant enzyme activities and reducing MDA production in immunosuppressed mice. Furthermore, HP2-1 potentiated immune responses both in vitro and in vivo by modulating the TLR4/MAPK/NF-κB pathway due to upregulating TLR4 expression, leading to phosphorylation of ERK, JNK, and p38 MAPKs, thereby activating NF-κB and subsequent cytokine secretion.

## 1. Introduction

The immune system plays a critical role in the defence of the host against microbes and external pathogens, immune system dysfunction could lead to inflammation, infection, and other diseases occur [1]. Hence, adequate immune function is essential to sustaining optimal health. Researchers have demonstrated that plant polysaccharides exert immunomodulatory effects by eliciting immune cell activation and thus promoting cytokine production [2]. *Ziziphus jujuba*, commonly known as the Chinese date, belongs to the genus *Ziziphus* in the family Rhamnaceae. It is a widely distributed species native to South Asia, East Asia, and Australia [3,4]. *Ziziphus jujuba* is rich in vitamin C, phenolic acids, polysaccharides, flavonoids, triterpenoids, cyclic nucleotides, and amino acids [5]. Wei et al. reported that porphyrin-derived oligosaccharides showed immunomodulatory activity, modulating the production of immunoglobulin G1 (IgG1), interleukin (IL)-2, interferon (IFN)-γ, and IL-17 in ovalbumin-sensitized mice [6]. Hu et al. extracted polysaccharides from different varieties of quinoa leaves, which promoted the capability to multiply RAW264.7 cells and produce nitric oxide (NO), IL-6, and tumour necrosis factor (TNF)-α [7]. Qiao et al. demonstrated that neutral polysaccharides from Sparassis latifolia promoted the growth of RAW264.7 cells, leading to the production of NO, TNF-α, and IL-6, and upregulated the expression of the immune receptor Toll-like receptor (TLR) 4 [8].

Polysaccharides interact with membrane receptors and initiate downstream signal transduction to regulate immune cell activation and function [9]. As key pattern recognition receptors, TLRs recognize diverse ligands and activate the mitogen-activated protein kinase (MAPK)/nuclear factor (NF)-κB signalling pathway through a MyD88-mediated cascade, thereby promoting cytokine expression and triggering immune responses [10,11]. Activation of the MAPK pathway, marked by increased phosphorylation of JNK, ERK, and 38, triggers the nuclear translocation of transcription factors, thereby regulating the expression of various cytokine genes [12]. Moreover, JNK, ERK, and p38 can phosphorylate the inhibitor of nuclear factor (NF)-κB (IκB) to dissociate NF-κB from IκB, thereby facilitating the induction of NF-κB transcription factors [13], which regulate the production of various cytokines associated with immunomodulatory effects, including TNF-α, IL-1β, and IL-6 [14]. Therefore, the MAPK/NF-κB pathway plays a pivotal role in the modulation of immune responses and the preservation of immune homeostasis. Polysaccharides derived from Sarcodon aspratus have been shown to activate RAW264.7 cells by inducting the NF-κB and MAPK pathways in a process mediated by TLR4, thereby inducing an immune response [15]. Sulfated polysaccharides from Chinese yam were also shown to enhance the immune activity of RAW264.7 cells by promoting the expression of reactive oxygen species (ROS), NO, and cytokines via the TLR4/MAPK/NF-κB pathway [16]. Furthermore, polysaccharides from *Nizamuddinia zanardinii* activated RAW264.7 macrophages and natural killer (NK) cells through activation of the MAPK/NF-κB signalling pathway [17]. Ganoderma atrum polysaccharides induced ROS production in macrophages via the TLR4/ROS/PI3K/Akt/MAPK/NF-κB signalling pathway [18].

Jujuba “Huizao” is one of the primary dried fruits in Xinjiang, China. In this study, “Huizao” polysaccharide (HP2-1) was extracted and isolated using DEAE Cellulose-52 and Sephadex G-100 chromatography. The immunomodulatory effects of HP2-1 on macrophages were evaluated by evaluating cell viability, phagocytic activity, and the production of immune molecules, including TNF-α, IL-6, IL-1β, and NO. The immunomodulatory effects of HP2-1 in immunosuppressed mice were also investigated by evaluating immune organ indices, morphology of the spleen tissue, cytokine levels in the spleen, immunoglobulin content in serum, and oxidative stress index in the liver. This study focused on exploring the immunomodulatory mechanisms of HP2-1 in vitro and in vivo, and will provide a foundation for the development of active ingredients in jujube.

## 2. Materials and Methods

### 2.1. Materials and Chemicals

Jujuba “Huizao” was purchased from Ruoqiang (Xinjiang, China). After removing broken and diseased seeds, “Huizao” was dried for 3 h at 60 °C and ground to a powder using a grinder; then, the powder was screened using a 40-mesh filter and stored at 4 °C for later use. RAW264.7 cells were purchased from the Cellverse Co., Ltd. (Shanghai, China) in liquid nitrogen preservation.

Diethylaminoethyl (DEAE)-52 cellulose, lipopolysaccharide (LPS), a cell counting kit (Neutral Red method), an ROS kit, and Sephadex G-100 were obtained from Solarbio (Beijing, China). Cyclophosphamide (CTX) was purchased from Sigma Co. (Roedermark, Germany). Fetal bovine serum (FBS) was obtained from Zhejiang Tianhang Biotechnology (Huzhou, China). DMEM was obtained from Thermo Fisher Scientific (Hangzhou, China). All other chemicals and reagents were commercially available and of analytical grade.

### 2.2. Extraction and Purification of Polysaccharides

“Huizao” polysaccharide (HP) extraction was performed as described previously [19] with modifications. In brief, the powder was defatted by homogenization with petroleum ether with a solid/liquid ratio of 1:10 g/mL at room temperature for 24 h. Then, degreased jujube lyophilizate (50 g) was mixed with distilled water (1.5 L) by ultrasonication for 20 min and heated at 70 °C for 3 h, followed by centrifuging to obtain the supernatant. The supernatant was evaporated and concentrated to 1/3rd, then treated with 4 times the volume of 95% ethanol overnight for precipitation. The precipitation was resolved, and the solution was mixed with D101 resin 1:4 (g/mL) to decolor, then deproteinized by adding the Sevag reagent (Chloroform:N-butanol, 4:1). The crude polysaccharide solution at a level of 30 mg/mL was uploaded onto a pre-equilibrated DEAE-52 cellulose anion exchange column (2.6 × 60 cm). Sodium chloride solutions, from low to high (0, 0.1 M, 0.3 M), were washed at 1 mL/min. Based on absorbance at 490 nm, the polysaccharide fractions were collected, then evaporated and freeze-dried. The lyophilized powder was put into a 5 mg/mL solution, and the solution was loaded to a Sephadex G-100 gel column (1.6 × 90 cm) and washed with deionized (DI) water at 0.3 mL/min. The resulting fractions (1 g of HP2-1 contains 938.37 mg of polysaccharide) were acquired, then evaporated and freeze-dried for further use.

### 2.3. Cell Viability and Phagocytic Activity Analysis

The viability of RAW264.7 cells was evaluated using the methylthiazolyldiphenyl-tetrazolium bromide assay (MTT). A cell suspension containing 1 × 10^5^ cells/mL was prepared, and 100 μL of the suspension was inoculated into 96-well plates. Next, 100 µL of LPS (1 µg/mL) and HP2-1 (25, 50, or 100 µg/mL) were added to the cell culture dishes. After culturing for 24 h, 100 µL of MTT solution was added to the culture medium, and the absorbance values were recorded using an ultraviolet spectrophotometer at 490 nm. After polysaccharide treatment, the phagocytic capacity of RAW264.7 cells was determined according to the kit instructions.

### 2.4. Measurement of Intracellular ROS Accumulation and NO Secretion

After 24 h of treatment with HP2-1 and LPS, 10 μmol/L of 2′-7′-dichlorodihydrofluorescein diacetate (DCFH-DA) was added to the culture medium for 30 min at 37 °C in a light-protected incubator. The fluorescence level was observed using an inverted fluorescence microscope. After treatment with HP2-1 and LPS for 24 h, the supernatants were collected. NO content was determined using a Griess kit (Biyuntian, Shanghai, China).

### 2.5. Animal Experiments

Sixty male BALB/c mice (20 ± 2 g; age, 6–8 weeks) were purchased from the Animal Experiment Center of Xinjiang Medical University (Animal Production Licence No.: SCXK (Xin) 2023-0001). The experimental protocols were authorized by the Tab of Animal Experimental Ethical Inspection of Laboratory Animal Centre, Xinjiang University (Approval number: XJUAE-2024-010). The mice were housed in a clean-level animal facility and fed a normal chow diet, and water was freely available during the experimental period. The facility was maintained at 23 °C–25 °C and 55–60% humidity, with a light–dark cycle of 12/12 h. Following a one-week acclimatization period, the animal experiments were initiated.

**Figure 1:**
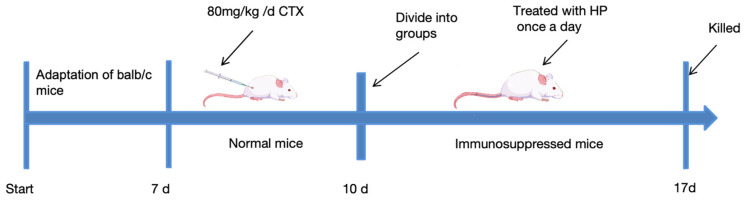
Animal experimental protocol and design.

As shown in Figure 1, the immunosuppressed mouse model was established as follows: normal control (NC) mice were injected with 0.9% normal saline, and mice in the other groups received intraperitoneal injections of 80 mg/kg CTX (16 mg/mL) once a day for 3 days. The immunosuppressed mice were divided into five random groups (*n* = 10, per group) for subsequent experimental treatments. For HP treatment, immunosuppressed mice were intragastrically administered 50 (HP-L), 100 (HP-M), or 150 (HP-H) mg/kg HP (1 g of HP contained 863.9430 mg of polysaccharides) intragastrically (IG) once a day for one week. The positive control (PC) group received 10 mg/kg levamisole hydrochloride (LH), while the model control (MC) and NC groups received 0.9% normal saline via gavage. All mice were fed normal chow throughout the experimental period. One week after dosing, all mice were fasted for 12 h.

### 2.6. Immune Organ Index Measurement and Histological Observation

After the final dose, the mice were weighed and euthanised by cervical dislocation. The spleens and livers were immediately isolated and weighed, and the immune organ index was computed as follows: organ index = organ weight (mg) × body weight (g).

Spleen tissues were excised and immediately placed in cold saline. Following fixation in 10% buffered formalin, the tissues were embedded in paraffin. Sections were sectioned at 4 µm using a microtome, and after staining the sections with haematoxylin and eosin (HE), the histomorphology of the spleen was observed using a laser confocal scanning microscope.

### 2.7. Evaluation of Oxidative Stress

Superoxide dismutase (SOD), catalase (CAT), and glutathione peroxidase (GSH-Px) activities, as well as malondialdehyde (MDA) levels, were evaluated in liver homogenates using assay kits from Beyotime Biotechnology (Shanghai, China).

### 2.8. Measurements of Cytokines, Immunoglobulins, and Phosphorylated Proteins

Following the manufacturer’s protocol, the levels of TNF-α, IL-6, and IL-1β were quantified, and the concentrations of immunoglobulins A (IgA) and immunoglobulins M (IgM) were determined using ELISA kits from Elabscience Biotechnology (Wuhan, China). And cytokine content was evaluated by standard protein concentration. TLR4 and the phosphorylation of Erk1/2, JNK, p38, and NFkBp65 were evaluated using ELISA kits from Meimian Industrial Limited (Yancheng, China).

### 2.9. Statistical Analysis

All results were presented as the mean ± standard deviation (SD). Intergroup differences were assessed by one-way ANOVA in GraphPad Prism 9, with statistical significance set at *p* < 0.05. Significant differences among groups are denoted by different lowercase letters.

## 3. Results and Discussion

### 3.1. Isolation and Purification of Polysaccharides

**Figure 2:**
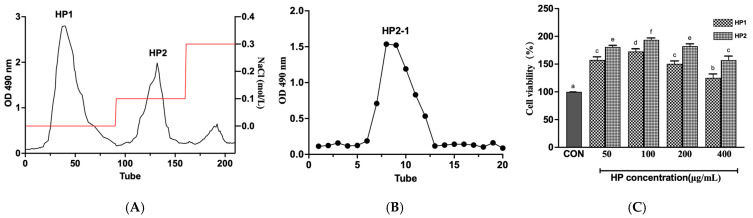
Isolation and purification of HP2-1. (**A**) DEAE-52 chromatography, (**B**) Sephadex G-100 chromatography, and (**C**) RAW264.7 cell viability analysis. a–f represent significant differences (*p *< 0.05).

As shown in Figure 2A, “Huizao” polysaccharides were firstly separated into two fractions (HP1 and HP2) using a DEAE cellulose-52 column. Both HP2 and HP1 significantly increased the proliferation of RAW264.7 cells, and HP2 caused greater enhancement than HP1 (Figure 2C). Therefore, HP2 was targeted for downstream purification using a Sephadex G-100 column, and HP2-1 exhibited a distinct single peak representing HP2-1 (Figure 2B).

### 3.2. Effects of HP2-1 on Cell Viability and Phagocytic Activity

**Figure 3:**
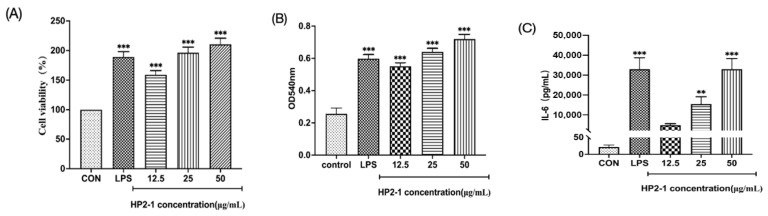

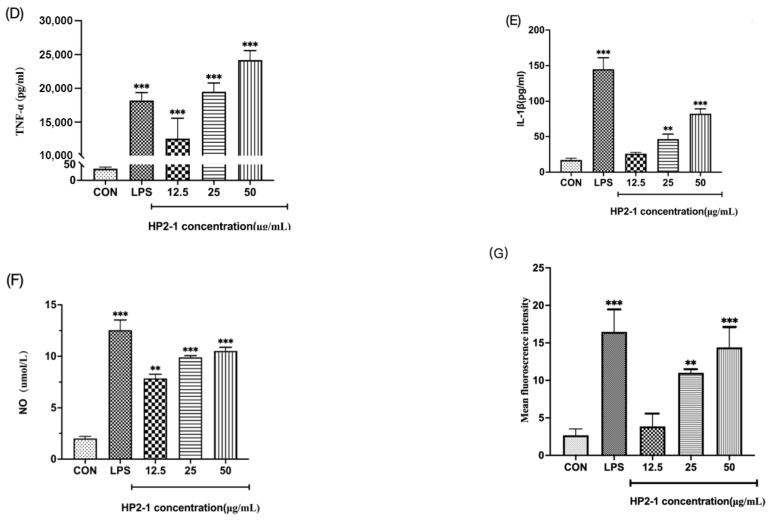
Effects of HP2-1 on the immunomodulatory activity of RAW264.7 cells ((**A**) cell viability, (**B**) phagocytic capacity, (**C**) IL-6, (**D**) TNF-α, (**E**) IL-1β, (**F**) NO, (**G**) ROS level). Note: in comparison with control **: *p* < 0.01; ***: *p* < 0.001.

The effects of HP2-1 on cell viability are shown in Figure 3A. At a concentration range of 12.5–50 μg/mL, HP2-1 had no effect on the normal growth of RAW264.7 cells, exhibiting no cytotoxic effects. Moreover, HP2-1 treatment resulted in a significant increase in the viability of RAW264.7 cells relative to the control (*p* < 0.05). Phagocytosis by macrophages is a commonly used indicator of the activation of immune function [20]. HP2-1 at the concentrations ranging from 12.5 to 50 µg/mL considerably promoted phagocytosis of RAW264.7 cells (*p* < 0.05) (Figure 3B), indicating that HP2-1 could increase the innate immune response by promoting the phagocytosis of RAW264.7 cells to invade pathogens. Li et al. investigated the immunomodulatory activity of a polysaccharide from ioscotea opposita, and found that the polysaccharide (10–500 μg/mL) enhanced the phagocytic activity of macrophage [21].

### 3.3. Effects of HP2-1 on Cytokines and NO Secretion

Macrophages kill pathogens both directly and indirectly through cytokine secretion and the regulation of immune homeostasis in the body’s environment [22]. In Figure 3C–F, HP2-1 (25–50 mg/mL) could significantly increase the production of the cytokines IL-6, IL-1β, and TNF-α (*p* < 0.05), and elevate NO accumulation (*p* < 0.05) in RAW264.7 cells. *Cystoseira indica* polysaccharides have been shown to considerably promote macrophages to release TNF-α, IL-1β, IL-6, and IL-10 [23]. Moreover, a sulfated polysaccharide from *Mesona chinensis* Benth was shown to significantly stimulate macrophages to promote the expression of IL-6, IL-1β, and TNF-α to exert immunomodulatory functions [24]. Sansone et al. found that the water-soluble non-starch polysaccharides from bananas displayed immunomodulatory properties on cultured macrophages [25]. The results suggested that HCP2-1 could promote the immunological activity of RAW264.7 cells by inducing the production of the inflammatory cytokines IL-6, IL-1β, and TNF-α and stimulating NO production.

### 3.4. Effects of HP2-1 on the Generation of ROS

ROS produced by macrophages and neutrophils are crucial antimicrobial factors that enhance inflammatory responses and promote the generation of cytokines in immune regulation [26]. As shown in Figure 3G, in comparison with the control group, RAW264.7 cells treated with HP2-1 showed significantly elevated intracellular green fluorescence intensity (*p* < 0.05). Wang et al. demonstrated that *Lepidium meyenii* Walp polysaccharides induced ROS secretion and displayed the immunomodulatory effects on macrophages [27]. The results indicated that HP2-1 promoted the accumulation of ROS in RAW264.7 cells and promoted the generation of pro-inflammatory cytokines.

### 3.5. HP2-1 Mediated TLR4/MAPK/NF-κB Signalling Pathway in RAW264.7 Cells

**Figure 4:**
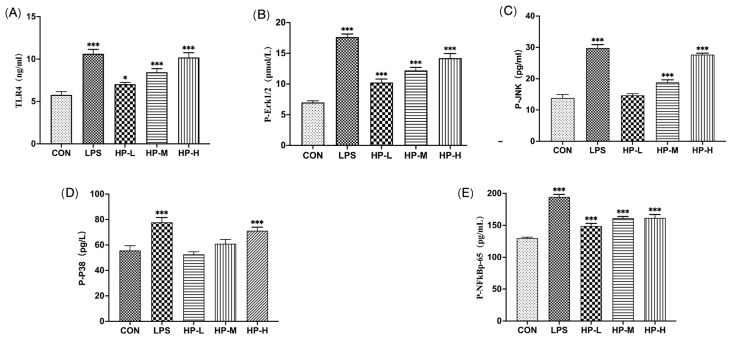
Effect of HP2-1 on protein expression in the TLR4/MAPK/NF-κB signalling pathway in macrophages. Note: in comparison with control *: *p* < 0.05; ***: *p* < 0.001. (**A**): TLR4, (**B**): P-ERK1/2, (**C**): P-JNK, (**D**): P-p38, (**E**): P-NF-κB p65.

The TLR4 immune receptor promotes the stimulation of JNK, ERK, and p38, which subsequently activate NF-κB transcription factors, facilitating the secretion of cytokines in the TLR4/MAPK/NF-κB signalling pathway. In Figure 4, HP2-1 significantly enhanced TLR4 expression and promoted the phosphorylation of JNK, ERK1/2, and p38 (*p* < 0.05) in the MAPK signalling pathway. Additionally, HP2-1 significantly increased the production of phosphorylated NF-κB p65 (*p* < 0.01), an important transcription factor associated with immunomodulatory effects. A previous study showed that the polysaccharide CPE-II activated macrophages through TLR4 and TLR2, and promoted IL-6 by regulating NF-κB and MAPKs [28]. Similarly, the polysaccharide from freshwater *M. spicatum* L. also activated RAW264.7 cells via MAPK and NF-κB signalling pathways [29]. Our results indicate that RAW264.7 cells were activated by HP2-1 through regulation of the MAPK/TLR4/NF-κB signalling pathway by increasing the expression of the immune receptor TLR4 and activating the JNK, ERK, p38, and NF-κB signalling pathways.

### 3.6. Effects of HP2-1 on Immune Organ Index

In this study, after three days of CTX administration, mice displaying weight loss and reduced appetite were selected for the experiments and subsequently allocated into groups. As illustrated in Table 1, the administration of CTX was observed to result in a notable increase in both the spleen and liver indices in BALB/c mice, indicating that CTX led to hepatic and splenic swelling. Administration of HP2-1 was followed by a substantial reduction in the spleen and liver indices (*p* < 0.05), suggesting that HP2-1 could promote immune activity in immunosuppressed mice by alleviating damage to immune organs.

### 3.7. Histological Observation of the Spleen

**Figure 5:**
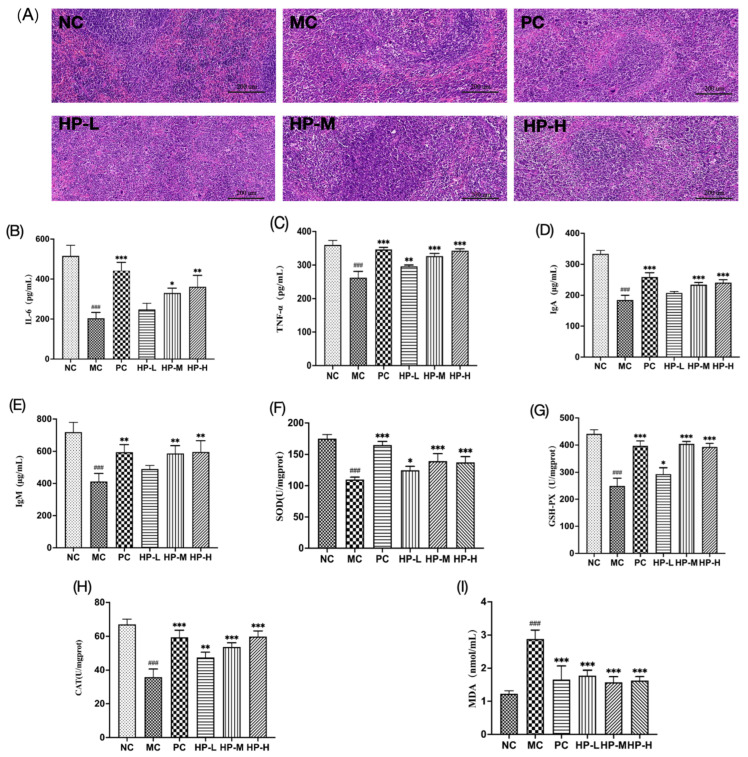
Effects of Jujube polysaccharides on the immunomodulatory activity in mice with cyclophosphamide-induced immunosuppression. (**A**) Histological findings for spleen tissue, (**B**) IL-6, (**C**) TNF-α, (**D**) IgA, (**E**) IgM, (**F**) SOD, (**G**) CAT, (**H**) GSH-PX, and (**I**) MDA. Note: in comparison with NC ###: *p* < 0.001. in comparison with MC *: *p* < 0.05; **: *p* < 0.01; ***: *p* < 0.001.

As a critical site for initiating immune responses and filtering blood, the spleen acts to preserve systemic immune homeostasis [30]. As shown in Figure 5A, the histopathology of the spleen was examined, and the spleens in normal control (NC) mice displayed a uniform distribution of red and white pulp with clear boundaries, orderly and densely arranged cells, and minimal gaps. In the model group, the interface between the white and red pulp appeared obscured, the white pulp area was reduced and scattered, and the cells were sparsely and irregularly arranged; the HE-stained histopathological images for this group revealed the presence of necrotic areas devoid of cellular structures. The HP-M and HP-H groups showed a relatively uniform distribution of red and white pulp, clearer boundaries, larger white pulp areas, and a more orderly cell arrangement, which were comparable to the findings in the control group. These findings indicate that HP2-1 significantly promotes spleen recovery in mice with CTX-induced immunosuppression.

### 3.8. Cytokine Production in the Spleen

Cytokines, which are known as biological response modifiers, include TNF and ILs, which regulate inflammation, immune responses, and defence against external pathogens [31]. The effects of HP2-1 on cytokine production are shown in Figure 5B,C. In comparison with NC, the production levels of TNF-α and IL-6 in the spleen tissue of the MC mice were significantly reduced (*p* < 0.05). After treatment with HP2-1, the production of cytokines TNF-α and IL-6 was significantly promoted in comparison with that in the immunosuppressed mice (*p* < 0.01). The polysaccharide extracted from Kadasura marmorata has been shown to enhance the immunoregulatory effects of the organism by increasing the production of IFN-γ, IL-2, and TNF-α from spleen-derived lymphocytes in culture [32]. Thus, HP2-1 may mediate immunomodulatory activity by upregulating the secretion of inflammatory cytokines in immunosuppressed mice.

### 3.9. Serum Levels of IgA and IgM

Immunoglobulins served as a crucial intermediate between adaptive and innate immunity, facilitating the direct clearance of pathogens and increasing the overall immune response by regulating the functions of various immune cells [33]. In Figure 5D,E, a significant reduction in serum IgA and IgM levels was observed in mice of the MC group versus the NC group. (*p* < 0.05), thereby substantiating the hypothesis that CTX can exert a considerable inhibitory effect on immunoregulatory function in mice. After administration of HP2-1, the moderate- and high-dose arms of jujube polysaccharides were able to significantly increase IgA and IgM levels in immunosuppressed mice relative to MC (*p* < 0.05). Huang et al. demonstrated that Chinese yam polysaccharide (CYP) can enhance immunomodulation of the body by promoting the production of immunoglobulins [34]. These results suggest that HP2-1 enhances immune function in immunosuppressed mice by promoting IgA and IgM secretions.

### 3.10. HP2-1 Attenuated Oxidative Stress

Abnormal immune responses have been shown to be associated with oxidative stress, which can lead to a decline in immune cell function [35]. As illustrated in Figure 5F–I, MDA accumulation in the liver tissue of immunosuppressed mice was markedly diminished by HP2-1 treatment (*p* < 0.01), whereas the activities of GSH-Px, SOD, and CAT were notably elevated (*p* < 0.05). These results suggested that HP2-1 ameliorated oxidative stress and consequently restored immune function in immunosuppressed mice.

### 3.11. HP2-1 Regulated the TLR4/MAPK/NF-kB Pathway in Mice

**Figure 6:**
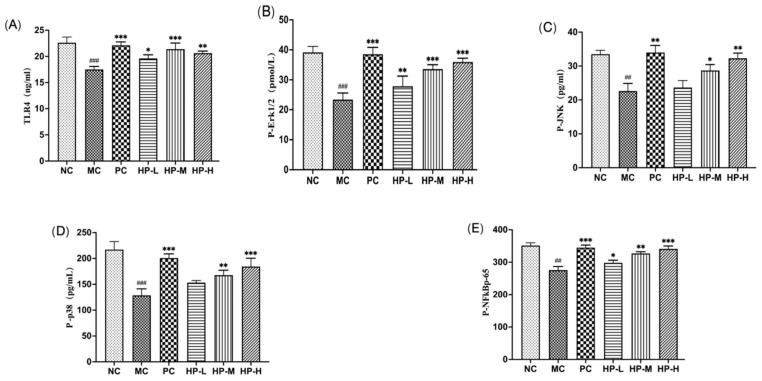
Effects of HP2-1 on protein expression in the TLR4/MAPK/NF-κB signalling pathway in CTX-treated mice. Note: in comparison with NC ##: *p* < 0.01; ###: *p* < 0.001. in comparison with MC *: *p* < 0.05; **: *p* < 0.01; ***: *p* < 0.001. (**A**): TLR4, (**B**): P-ERK1/2, (**C**): P-JNK, (**D**): P-p38, (**E**): P-NF-κB p65.

As illustrated in Figure 6, a notable decline in TLR4 levels was observed in the spleen of the model control (MC) group in comparison with the (normal control) NC group (*p* < 0.01). Additionally, a significant reduction was evident in the phosphorylation levels of JNK, ERK, p38, and NF-κB p65 in the MAPK pathway (*p* < 0.01). After treatment with HP2-1, TLR4 expression increased significantly (*p* < 0.05), and a pronounced increase was observed in the phosphorylation levels of ERK1/2, p38, and JNK in immunosuppressed mice (*p* < 0.05), while the expression of phosphorylated NF-κBp65 also increased significantly (*p* < 0.01). Meng found that polysaccharides obtained from the mycelia of Cordyceps gunnii demonstrated immunomodulatory activity through the TRAF6/TLR4/NF-κB signalling pathway [36]. Li et al. reported that astragalus polysaccharide treatment significantly activated the TLR4 and MAPK pathways in the intestine [37]. Our results elucidated that immune activation in immunosuppressed mice was elevated by HP2-1 through modulation of the MAPK/TLR4/NF-κB signalling pathway by increasing expression of the immune receptor TLR4 and enhancing the phosphorylation of p38, JNK, and Erk1/2, as well as the NF-κBp65 transcription factor.

## 4. Conclusions

In conclusion, “*Huizao*” polysaccharides (HP2-1) promoted the phagocytic capacity of RAW264.7 cells and increased the secretion of the cytokines IL-6 and TNF-α and ROS production. HP2-1 also improved immune competence via boosting IgA and IgM production in the serum of immunosuppressed mice. Additionally, HP2-1 alleviated oxidative stress by increasing the antioxidant enzyme activities of GSH-Px, CAT, and SOD, while reducing MDA expression in the liver. Furthermore, HP2-1 enhanced immune responses in RAW264.7 cells and in immunosuppressed mice by modulating the MAPK/TLR4/NF-κB pathway, leading to an increase in immune receptor TLR4 expression, promoting the activation of nd p38 in the MAPK signalling pathway, and activating NF-κB transcription factors to facilitate the secretion of cytokines. In summary, HP2-1 could be used as a natural product for strengthening immune capacity, and highlighted its application as a potential component in functional food.

## Figures and Tables

**Table 1:** Effects of HP2-1 on immune organ indices in immunosuppressed mice.

Groups	Spleen Index (mg/g)	Liver Index (mg/g)
NC	3.8216 ± 0.15796 ^d^	54.2475 ± 0.71141 ^e^
MC	6.7031 ± 0.11768 ^a^	54.2475 ± 0.71141 ^e^
PC	5.1227 ± 0.10677 ^c^	62.1002 ± 1.53651 ^bc^
HP-L	6.0136 ± 0.10361 ^b^	68.8005 ± 2.28967 ^b^
HP-M	5.8277 ± 0.15346 ^b^	63.7642 ± 1.0463 ^c^
HP-H	5.8145 ± 0.2246 ^b^	61.1067 ± 1.78673 ^d^

Different letters for the same index among groups indicate significant differences at *p* < 0.05.

## Data Availability

The original contributions presented in this study are included in the article. Further inquiries can be directed to the corresponding author.
